# Determinants of Hypertension amongst Rice Farmers in West Java, Indonesia

**DOI:** 10.3390/ijerph19031152

**Published:** 2022-01-20

**Authors:** Nurhayati Adnan Prihartono, Laila Fitria, Doni Hikmat Ramdhan, Fitriyani Fitriyani, Sifa Fauzia, Susan Woskie

**Affiliations:** 1 Department of Epidemiology, Faculty of Public Health, Universitas Indonesia, Depok 16424, Indonesia; fitriyani.sukesmi@gmail.com; 2 Department of Environmental Health, Faculty of Public Health, Universitas Indonesia, Depok 16424, Indonesia; lfitria@ui.ac.id (L.F.); sifafauzia@ui.ac.id (S.F.); 3 Department of Occupational Health and Safety, Faculty of Public Health, Universitas Indonesia, Depok 16424, Indonesia; doni@ui.ac.id; 4 Department of Public Health, University of Massachusetts Lowell, One University Ave, Lowell, MA 01854-2867, USA; susan_woskie@uml.edu

**Keywords:** hypertension, farmer, occupational factor, heat

## Abstract

The hypertension rate in Indonesia has increased significantly in the past five years, but there is limited information about the hypertension risk of farmers. Our study assesses the prevalence of hypertension in this population and examines the proportional risk of various work environment and lifestyle factors among farmers. A cross-sectional study was conducted in high and low heat stress agriculture areas of West Java, Indonesia. There were 354 male farmers aged 25 to 73 years old who participated in the study. We measured blood pressure and used a questionnaire on self-reported use of anti-hypertension drugs or diagnosis by a medical professional to define hypertension. We assessed occupational factors including farming methods, heat stress and pesticide use, and personal factors including obesity, food intake, smoking status, alcohol consumption. Multivariate analysis was used to evaluate factors potentially associated with prevalence of hypertension. Forty-six percent of farmers experience hypertension. Farming in a location with higher heat stress (WBGT) was significantly associated with increased risk of hypertension (adjusted prevalence ratio (aPR) 1.41, 95% confidence interval (CI) 1.01, 1.95). Farmers who used pesticide sprayers had an increased risk of hypertension (aPR 1.90, 95% CI 0.93, 3.87). No personal/lifestyle characteristics were significantly associated with hypertension, although ever smoking and ever consuming alcohol had an increased prevalence of hypertension. This study shows the importance of work environmental factors in the prevalence of hypertension and the necessity of public health education, identification and treatment of this “silent killer” among Indonesian farmers.

## 1. Introduction

Hypertension is still a health problem in low-middle income countries including Indonesia [1,2,3]. Based on the Indonesian House Health Survey conducted in 2018, the prevalence of hypertension based on a doctor’s diagnosis among those aged 18 years and older was 8.36% [4]. According to population screening surveys, the prevalence of hypertension from measurement was much higher than only those with a doctor’s diagnosis, with 34.11% hypertensive in 2018 compared to 25.8% in 2013 from measurement [4,5]. This suggests there is a growing fraction of the population who are unaware that they have hypertension [4,5]. The prevalence of hypertension in West Java is slightly higher than the national figure (39.60% in 2018), with West Java having the second highest prevalence of hypertension among all provinces in Indonesia [4].

It has been known that hypertension is generally believe to be a major risk factor for cardiovascular diseases (CVD), kidney disease, metabolic syndrome, and also a cause of premature death. Typically, there are no symptoms, therefore hypertension is called a silent killer. Research on risk factors for hypertension has been widely conducted including examinations of the role of obesity, smoking, alcohol consumption, unhealthy diet, and sedentary lifestyle [1,6,7,8]. In addition to the behavioral and lifestyle risk factors, occupation is another risk factor that could be associated with hypertension. Previous studies found that hypertension is associated with physical workload, shiftwork, and long working hours [9,10]. CVD associated with hypertension has been associated with exposure to certain chemicals, long working hours, noise, and stress [11]. Several previous studies stated that the prevalence of hypertension and CVD was higher in farmers than non-farmers [12,13]. Research on hypertension among farmers in China found a high prevalence of hypertension and a relationship with increasing age, gender, higher body mass index (BMI), physical activity, and diet [14]. Another study found hypertension to be associated with increasing age and BMI, but no relationship to alcohol, tobacco consumption, and household size as a proxy of stress in the population of an agricultural area of Madagascar [15]. Data from the Indonesian House Health Survey conducted in 2018 found that the prevalence of hypertension in farmers/laborers/fisherman tends to be higher than in other occupations [4]. However, in Thailand, unskilled workers were found to have a significantly higher risk for hypertension compared to agricultural workers [16], and there was not significant difference in rates of abnormal blood pressure between organic and conventional (pesticide using) farmers [17].

Given these contradictory findings, this study was designed to investigate the prevalence of hypertension in West Java rice farmers and to explore the work environment and lifestyle factors associated with higher risk of this disease. 

## 2. Materials and Methods

This field study was conducted from 29 June to 8 July 2018 to investigate the adverse health effects of occupational, environmental, and as well as life-style factors among rice farmers in West Java, Indonesia. Previous study results have been published on the prevalence and risk factors for chronic kidney disease of unknown origin [18]. Briefly, we describe the study methods.

### 2.1. Ethical Clearance

The study has been reviewed by The Research and Ethics Committee of Faculty of Public Health, Universitas Indonesia (letter number 73/UN2.F10/PPM.00.02/2018, March 6, 2018 and number 467/UN2.F10.D11/PPM.00.02/2021, 7 September 2021). 

### 2.2. Study Design

The study focused on male rice farmers in West Java, Indonesia. The study design was cross-sectional, including two different geographic locations in West Java, i.e., Karawang Regency and Bogor Regency. Both Karawang and Bogor are the largest rice paddy areas in Indonesia, but the two-study locations differed in altitude and climate. Karawang Regency is located in a low altitude region at about 0–5 m above sea level, which has a warmer climate, while Bogor Regency is located in a higher altitude region about 450 m above sea level and has a cooler climate. From each regency, we selected one village that had a large farmer population: Sukamerta Village in Karawang Regency and Jambuluwuk Village in Bogor Regency.

### 2.3. Population and Sample

The study population was adult male rice farmers between the ages of 20 through 65 years who currently live in the village. We used the household list from the village office (in Karawang) and the farmers list from the head of the farmers group (in Bogor) as the sampling frame to recruit the study subjects (male farmers) using a systematic random sampling method. The determination of the sample size followed the calculation of the sample size in previously conducted studies related to chronic kidney disease (CKD) in rice farmers. The samples obtained amounted to 186 farmers in the Sukamerta Village, Karawang Regency and 168 farmers in the Jambuluwuk Village, Bogor Regency. Thus, the overall sample size was 354 farmers. In this study, we included all the study subjects recruited to analyze hypertension and its risk factors.

### 2.4. Data Collection Procedures

There were three teams that were involved in data collection in each village and they worked at the same time. One team consisted of five interviewers who went house-to-house to recruit study participants and collect data using a questionnaire; another team consisted of laboratory staff who collected blood samples, anthropometry, and blood pressure measurements from participants; and a third environmental team measured ambient temperature and relative humidity, as well as air velocity in rice fields.

#### 2.4.1. Assessment of Resting Blood Pressure

One of the assessments in this study was systolic and diastolic blood pressure measurement and determination of normal vs. hypertension status [19]. Blood pressure measurements were obtained by trained field coordinators using a validated digital sphygmomanometer device (Omron Automatic Blood Pressure Monitor HEM 7156, Kyoto, Japan) and measured blood pressure in left arm. Study participants waited in line before the initial measurement. Respondents quietly sat in a reclined chair with both feet flat on the floor, legs uncrossed, the forearm position rested on a table and flexed at the elbow at the level of the heart. Blood pressure measurements were carried out twice with a resting interval of 1–2 min, and the average was calculated and recorded for data analysis [19].

#### 2.4.2. Assessment of Hypertension Risk Factors 

Data on hypertension risk factors was collected by interviewers who had been trained in the use of our questionnaire. Questions included age, marital status, educational level, income, smoking status and alcohol consumption as well as occupational risk factors (duration of farming, farming methods, pesticides used, duration of employment, duration of pesticide use, whether a pesticide sprayer). Diet information was collected using a questionnaire with a semi-quantitative method on each type of food such as vegetables, fruits, red meat, high fat meat and salted fish or squid consumption. The questionnaire used in this study was developed in accordance with the recommendations of the DEGREE Core Protocol [20]. The BMI variable was collected by trained field coordinators who made anthropometric measurements (height and weight). Body Mass Index was calculated as body weight in kilograms divided by height in meters squared. Non-fasting blood sugar was measured from venous blood specimen that was taken at 09.00–11.00 in the morning. The blood specimen then immediately transported to the laboratory and analyzed in the same day using the Hexokinase method with Proline reagent, in an automated clinical analyzer TMS 24i Premium (Tokyo Boeki Medisys Inc. Tokyo, Japan). 

In the questionnaire, we also determined the farming methods practiced by farmers. Farming method was categorized as modern farming if the farmers practiced farming dominantly using mechanical equipment such as a tractor, transplanter, combine harvester, and other mechanical equipment. The farming method was categorized as traditional farming if the farmers practiced farming dominantly using simple manual equipment such as hoes, machetes, sickles, harrows, and buffalo to plow the fields. If the farmers practiced farming using both mechanical equipment and manual equipment equally, then the farming method was categorized as mixed. 

We measured ambient temperature and relative humidity in the rice field every day during the whole week of the data collection. The Wet Bulb Globe Temperature (WBGT) was recorded every hour for the rice fields’ entire working day, starting from 6 a.m. to 6 p.m. using a QUESTemp 34 Thermal Environmental Monitor (Quest Technologies, Oconomowoc, WI, USA). Furthermore, this WBGT data was used to determine heat stress exposure to farmers.

### 2.5. Data Processing

A study subject was categorized as hypertensive if their averaged blood pressure was ≥140 systolic and/or diastolic ≥ 90 [19] and/or they self-reported that they were currently taking antihypertensive medicine in the last three months; otherwise, the study subject was categorized as normal. In this study, all but one subject who were currently taking antihypertensive medicine having blood pressure ≥ 140 systolic and/or diastolic ≥ 90. The self-reported declared hypertension by a doctor or health worker was not included for the analysis because 19.5% of the respondents stated do not know whether they are having hypertension. To examine the risk factors of hypertension in our study areas, we stratified the prevalence of hypertension by age, marital status, income, educational level (low if junior high school or less and high if senior high school or above). Income information was obtained by the amount of income per month (absolute number). Income variable was categorized based on population income by Central Bureau of Statistic Indonesia, namely group 1 or low (IDR ≤ 1,500,000), group 2 or moderate (IDR > 1,500,000–2,500,000), group 3 or high (IDR > 2,500,000–3,500,000) and group 4 or very high (IDR > 3,500,000) [21]. We treated the income as a continuous variable in the multivariate model. 

Body mass index was categorized according to the Indonesian BMI classification based on the WHO recommendations for Asia Pacific populations: underweight (BMI < 18.5 kg/m^2^), normal weight (18.5–24.9 kg/m^2^), overweight (25–29.9 kg/m^2^), and obesity (≥30 kg/m^2^) [22]. However, since the number of the underweight and obese was too low, we combined underweight and normal weight into (BMI < 18.5–24.9 kg/m^2^) and combined overweight with obese (BMI 25–≥30 kg/m^2^) for the analysis. Blood sugar was categorized as high if ≥200 mg/dl and normal if <200 mg/dl [23], smoking status was categorized as current, past or never; and alcohol consumption was categorized as ever/never since there were very few past and current alcohol consumers. 

For the diet variable, the frequency of consuming certain foods was measured through a questionnaire. For each type of food there were six choices and each answer was given a score, namely a score of 1 for the answer ‘never consume’, a score of 2 for the answer ‘1–3 times per month’, a score of 3 for the answer ‘1 day per week’, a score of 4 for the answer 2–4 times per week’, a score of 5 for the answer ‘5–6 times per week’, and a score of 6 for the answer ‘everyday’. In the data analysis, several categories of food were combined, namely red meat consumption was combined with high fat meat consumption, and vegetables consumption was combined with fruit consumption. The final categorization of the food frequency data for the meat and fruit/vegetable combined categories was determined by adding up the scores and then grouping them into 2 categories based on the median cut-off value, namely ‘frequent’ (if the score was ≥median) and ‘seldom’ (if the score was <median). For the consumption of salted fish or squid, the data was not combined, but as a unit of variable. Scores for consumption of salted fish or squid were also categorized into ‘frequent’ and ‘seldom’.

We estimated pesticide exposures using a questionnaire that included questions about the use various types of pesticide, duration of pesticide used, and whether the subject conducted mixing and application of pesticides. In our study, insecticides were the most commonly used pesticides in farming, while herbicides and fungicides were not commonly used in our study area. For this reason, we focused on insecticides for developing the exposure estimates. In determining the heat stress data, we used the WBGT data from ambient temperature and relative humidity measurement as in our previous study [17]. The WBGT measurement was taken in July 2018, and data showed that in Bogor Regency (25.3 ± 3.1 °C), the average WBGT was consistently lower than in Karawang Regency (29.6 ± 2.5 °C) [24]. This finding was strengthened by the long-term contextual information on average heat and humidity that is available from existing weather stations over a one-year period before the study (January–December 2017). The average heat and humidity data was obtained from the weather station in Bogor Regency. The average temperature and humidity were 21.5 °C and 85.9% for an average heat index of 22 °C, while for Karawang Regency (data obtained from the weather station in East Jakarta, that is the nearest weather station), the average temperature and humidity were 28 °C and 77% for a heat index of 32 °C [24]. Based on this combination of data sources, we categorized Bogor Regency as lower WBGT and Karawang Regency as higher WBGT. 

### 2.6. Statistical Analysis

To assess determinants for hypertension where the hypertension prevalence is high, we estimated prevalence ratio (PR) and 95% confidence interval (CI). We performed multivariate analysis with modified Cox Proportional Hazard Regression by assigning a constant risk period to calculate PR and 95% CI [25,26]. In addition, we assessed collinearity of all occupational variables (duration of farming, pesticide use, farming methods, duration of pesticide use, whether pesticide sprayer and heat stress/location) before entering variables into the multivariate analysis and found only working as a pesticide sprayer and pesticide use were collinear, therefore the variable of pesticide use was excluded from the multivariate analysis. Multivariate analysis was performed by including variables that have a *p*-Value ≤ 0.25 from the univariate analysis. Then, we used backward elimination to select those variables that significantly predicted hypertension (*p*-Value < 0.10) and also took into account the *p*-Value of Likelihood Ratio (LR) chi-square test of the model (<0.10). Two variables with significantly predicted hypertension were included (heat stress/WBGT/location and pesticide sprayer). Three other variables (smoking, alcohol consumption, and age) that were a priori related to hypertension regardless of significant level were retained in the final model [27,28,29].

## 3. Results

Among 354 farmers in this study, 46.6% had hypertension based on blood pressure measurements and/or taking medicine (46.3% based on measurements only, data were not shown) and 13% reported hypertension (Table 1). Mean levels of SBP (systole blood pressure) were 138.6 and mean levels of DBP (diastole blood pressure) were 83.9. Among the hypertensive farmers, the mean levels of systole and diastole blood pressure were 156.3 mmHg and 92.1 mmHg; while among the non-hypertensive farmers, the mean levels of systole and diastole blood pressure were 123.2 mmHg and 76.7 mmHg. Table 2 shows descriptive statistics of the study population and the prevalence of hypertension according to socio-demographic characteristics, lifestyle risk factors, and the health status of the farmers. Approximately 72% of the farmers were age 45 years or above. The prevalence of hypertension based on age category was similar, except for age 35 to 44 years where the prevalence of hypertension was slightly higher (53.5%). Nearly all farmers have a spouse. Those who have a spouse have a higher prevalence of hypertension (47.3%) than who do not (38.5%). The majority of farmers have lower education and lower income. There is no difference in the prevalence of hypertension between those who have lower education and higher education. Likewise, for income categories. 

Based on BMI, only a small number of farmers are either overweight or obese. Those farmers who are overweight or obese have a higher prevalence of hypertension (53.6%) compared to those with normal weight. Likewise, few farmers have a blood glucose level > 200 mg/dL, but there is no meaningful difference in hypertension prevalence by blood glucose category. Farmers who eat red meat or high fat meat, or salted fish or squid more frequently have a lower prevalence of hypertension than those farmers who eat these food groups less frequently. The prevalence of hypertension is slightly higher for farmers who eat fruit or vegetable less frequently compared to those farmers who eat this food group more frequently.

Most farmers smoke cigarettes. With 93% reporting ever smoked and 88% still smoke. The percentage of hypertension among past smokers, current smokers, and never smokers were 60.5%, 44.8%, and 46.2%, respectively (*p*-value 0.32). Drinking alcohol is not common in this population, approximately 10% of farmers report ever drinking alcohol. There was slightly higher prevalence of hypertension among farmers who ever drank alcohol (61%) as compared to those who never drank alcohol (45%).

As Table 3 shows, most of the famers used pesticides and also sprayed pesticides themselves. The percentage of farmers with hypertension is higher among those who used pesticide (47.9%) compared to who did not (27.3%). Likewise, farmers who sprayed the pesticides had a higher prevalence of hypertension (48.8%). Farming methods for this study varied, with majority using traditional or mixed methods (modern and traditional) and a small proportion using predominately modern methods. According to the methods of farming, farmers who used modern methods of farming have the highest prevalence of hypertension (60.5%), followed by those who used mixed methods (49.2%) with those using traditional methods having the lowest prevalence (38.3%) (*p*-value 0.048). The percentage of farmers with hypertension appeared to be constant according to categories of duration of farming and duration of pesticide use. However, farmers who reside in the higher heat stress/lower altitude region (Karawang) have a higher prevalence of hypertension (54.8%) compared with those who reside in in the lower heat stress/higher altitude region (Bogor) (36.5%).

Table 4 shows the relationship between hypertension and a range of risk factors from univariate and multivariable analyses. Univariate analysis shows two factors significantly associated with hypertension, farming methods (*p*-value 0.048) and heat stress/location (*p*-value 0.018). Being a pesticide sprayer increases the prevalence of hypertension but was of borderline significance (PR 1.95, 95% CI 0.96, 3.97, *p*-value 0.065). Other factors that were examined and found not to have a statistically significant association with hypertension included social-demographic factors, obesity, blood glucose, smoking, alcohol consumption, length of farming and length of pesticide use. In the final multivariable model, high heat stress (WBGT)/low altitude/living in Karawang was significantly associated with hypertension (aPR 1.46, 95% CI 1.07, 2.00). Being a pesticide sprayer increased the prevalence of hypertension about two-fold compared to being a non-sprayer (aPR 1.90, 95% CI 0.93, 3.87, *p*-value 0.070). There were no association between hypertension and smoking, alcohol consumption or age.

## 4. Discussion

The present study is the first study to investigate the occupational, environmental, as well as lifestyle risk factors for hypertension among farmers in Indonesia. This study found that about 46% of the farmers were classified as hypertensive, which is higher than the prevalence of hypertension in the general population of Indonesia, which was 34% in 2018. The hypertension prevalence we found in two villages in West Java was similar with the prevalence of hypertension among farmers in China and Madagascar [14,15], but higher than a study of farmers in Greece [12] and lower than a study of farmers in Australia [13]. The difference in hypertension prevalence may be related to differences in individual characteristics between the areas. For example, about two-thirds of farmers in Australia are classified as obese [29] compared to 15.8% of farmers overweight or obese in this study. It is hypothesized that adipocyte dysfunction in persons with obesity contributes to vascular and systemic insulin resistance and dysfunction of the sympathetic nervous system and the renin-angiotensin-aldosterone system [30]. Structural and functional changes in the kidney including activation of intra renal angiotensin II are also important in the development of obesity-associated hypertension [30]. This study also found that the majority of farmers are unaware that they have hypertension, with only 13% of farmers have been declared hypertensive by a doctor or health worker. This finding is consistent with a study by Yang et al., which found self-reported prevalence of hypertension at 15% in the US [10]. A marked difference in prevalence of hypertension obtained by self-report and by blood pressure measurement indicates that awareness about hypertension is still minimal among farmers in Indonesia. Other studies have also observed a high rate of undiagnosed hypertension in rural communities [31]. To understand why farmers have a higher prevalence of hypertension, we examined a range of potential demographic, lifestyle, and work environment risk factors for their association with hypertension. We found that a significant predictor of hypertension in farmers was their work location which also related to their potential heat stress (WBGT) during work. Farmers from Karawang were at significantly higher risk for hypertension. Karawang workers are under more heat stress due to the higher WBGT than farmers from Bogor which has a lower WBGT.

A study on sugarcane cutters with an increased risk of heat stress found a significant increase in heart rate, temperature, and blood pressure (SBP) occurred across the work shift [32]. However, another study found a significant increase in heat rate, but a decrease of SBP and DBP across 4 h shifts in sugarcane cutter in El Salvador [33]. During heat stress, thermo receptors will send information to the hypothalamus, resulting in two responses: vasodilation of skin blood vessels and sweating [34]. This vasodilation causes the heart to beat faster, increasing the amount of blood pumped to the skin to release core heat to the environment. Prolonged exposure to heat and sweating can reduce the amount of fluid in the body, reduce blood volume and cause dehydration [35]. However, chronic dehydration conditions can cause constriction of blood pressure. When the body’s cells lack water, the brain signals the pituitary gland to secrete vasopressin or antidiuretic hormone (ADH). Vasopressin (VP) constricts blood vessels via its action on V1 receptors and decreases urinary water excretion via V2 receptors [36].

Although not significant, our study showed that farmers using modern farming methods were at higher risk for hypertension compared with farmers using traditional farming methods. Most of the modern method farmers were in the high heat stress/WBGT region of Karawang. Modern farming methods use more mechanical equipment such as tractors, transplanters, and combine harvesters, while traditional farming methods are carried out using several types of simple manual equipment such as hoes and machetes, as well as buffalo to plow the fields. The use of modern equipment is less physical, but could affect blood pressure due to the noise resulting from the machines. Noise exposure may affect blood pressure by the mechanism of catecholamine depletion and the impairment of cardiac baroreceptor reflexes [37].

This study found that pesticide sprayers had a risk of hypertension 1.90 times higher than non-sprayers after controlling for other covariates. In this study, 94% of farmers reported using pesticides and 91% reported spraying those pesticides themselves. Samsudin et al., found a relationship between pesticide exposure among mosquito control workers and hypertension [38]. In comparisons of organic and conventional pesticide using farmers, use of pesticide was associated with increased risk of thyroid imbalances, higher lipid and glucose levels, higher BMI and waist circumference, and higher systolic and diastolic blood pressure [39]. Other studies have shown that pesticide exposure plays a role in the development of atherosclerosis and high blood pressure [40]. This might be due to pesticide-induced liver damage, leading to oxidative stress and hyperlipidemia [40]. Studies have shown that exposure to pesticides is associated with cardiovascular disease risk, with some hypothetical mechanism [41]. Evidence suggests that exposure to pesticides can alter thyroid hormone levels [17,42] and that pesticide exposures may be an important risk factor in increasing Thyroid Stimulating Hormone (TSH) levels, which in turn increases both systolic and diastolic blood pressure [43].

Our research was unable to identify the types of pesticides associated with hypertension, as farmers used many types of pesticides. When operating manual or motorized hand sprayers, farmers are exposed to significant amounts of the pesticide spray, which can enter the body through the skin and respiratory tract [44,45]. Pesticide handlers in paddy farms have reported symptoms associated with organophosphate and carbamate pesticide exposures such as difficulty in concentrating, numbness, excessive sweating, skin itchiness and slower body movements [46]. Exposure to organophosphates, a common type of pesticide that is widely used in agriculture, could affect the neurotransmitter acetylcholine by inhibiting the action of the enzyme cholinesterase, which results in an increase in the amount of acetylcholine. Higher acetylcholine at neuromuscular junctions affects muscular activity in the skeletal, cardiac and visceral muscle [41], which could alter blood pressure.

This study did not observe age as a predictor for hypertension. This might be due to the healthy survivor effect. More than half of the farmers (66%) have been working for long durations (10 years or more), and farming requires sufficient physical activity that can prevent high blood pressure.

One typical limitation of many cross-sectional studies comes from the attempt to establish a causal association with exposure status after people know they are sick and have changed their lifestyle exposure status (changed diet, smoking, alcohol consumption, etc.). This is an unlikely explanation for our inability to detect an association with lifestyle risk factors for hypertension in this study. First, the majority of farmers in this study did not know about their hypertension status, and second, we found that farmers who were overweight or obese, and had ever smoked or drunk alcohol had a slightly higher prevalence of hypertension (although non-significant). In addition, homogenous profiles in terms of low BMI and low education could be a study limitation to detect statistical association. A major concern of our study is the difficulty in measuring the food intake using a food frequency questionnaire for only one point in time. Food intake may have considerable variation over time. Therefore, it is very likely to result in substantial misclassification. A similar result was found in Indonesia Family Life Survey5 (IFLS-5), which did not observe an association between food intake and hypertension [47].

## 5. Conclusions

In summary, this study suggests that farmers in locations with higher WBGT/heat stress are at higher risk of hypertension. In addition, spraying pesticides increases the risk of hypertension. We found a high prevalence of hypertension among the farmers of West Java. As hypertension is known to put individuals at significant risk of other health effects, e.g., cardiovascular diseases, or death, a focus on developing educational and outreach programs to identify and treat hypertension among agricultural workers should be a public health priority.

## Figures and Tables

**Table 1:** Prevalence of hypertension based on blood pressure measurement (average of two measurements, 1–2 min apart) and/or self-report of currently taking antihypertension medicine.

Variables	n (%)	95% CI
Hypertension based on measurement and/or taking medicine		
Hypertension	165 (46.6)	41.3–51.8
No hypertension	189 (53.4)	48.1–58.6
Self report of being declared hypertension by doctor or health workers *		
Hypertension	46 (13.0)	9.5–16.5
No hypertension	239 (67.5)	62.6–72.4
Unknown	69 (19.5)	15.3–23.6
Systolic blood pressure (SBP) (mean, mmHg)	138.6	136.2–140.9
Diastolic blood pressure (DBP) (mean, mmHg)	83.9	82.6–85.2

Definition of hypertension: averaged blood pressure was ≥140 mm Hg systolic and/or diastolic ≥ 90 mm Hg and/or they self-reported that they were currently taking antihypertensive medicine. * Self report of being declared hypertension by doctor or health workers (did not included in the definition of the hypertension in further analysis).

**Table 2:** Prevalence of hypertension based on demographical, historical diseases, lifestyle and diet characteristics in Karawang and Bogor in West Java, Indonesia 2018.

Characteristics	Total (n = 354)	% Hypertension (n = 165)	95% CI	*p*-Value
Age (Years)				0.69
≥55	129	44.2	35.5–52.9	
45–54	129	46.5	37.8–55.2	
35–44	71	53.5	41.6–65.4	
<35	25	40.0	19.4–60.6	
Marital Status				0.53
Did not have spouse	26	38.5	18.4–58.5	
Have spouse	328	47.3	41.8–52.7	
Educational level				0.76
Low	326	46.9	41.5–52.4	
High	28	42.9	23.3–62.4	
Income (IDR)				0.81
≤1,500,000	185	44.9	37.6–52.09	
>1,500,000–2,500,000	80	50.0	38.8–61.2	
>2,500,000–3,500,000	45	48.9	33.7–64.1	
>3,500,000	44	45.5	30.1–60.8	
BMI (kg/m^2^)				0.406
25.0–≥30	56	53.6	40.1–67.1	
<18.5–24.9	298	45.3	39.6–50.9	
Non-fasting blood sugar				0.92
High (≥200 mg/dL)	9	44.4	39.3–84.9	
Normal	345	46.7	41.4–51.9	
Smoking status				
Ever	328	53.8	41.2–52.1	0.97
Current	290	44.8	39.1–50.6	
Past	38	60.5	44.2–76.8	
Never	26	46.2	25.6–66.7	
Alcohol consumption				0.18
Ever	34	61.8	44.6–78.9	
Never	320	45.0	39.5–50.5	
Red meat and high fat meat consumption				0.27
Frequent	204	43.1	36.3–49.9	
Seldom	150	51.3	43.2–59.4	
Fish or squid salted consumption				0.18
Frequent	186	41.9	34.8–49.1	
Seldom	168	51.8	44.2–59.4	
Fruits and vegetables consumption				0.71
Seldom	175	48.0	47.4–62.1	
Frequent	179	45.3	37.9–52.6	

Definition of hypertension: averaged blood pressure was ≥140 mm Hg systolic and/or diastolic ≥ 90 mm Hg and/or they self-reported that they were currently taking antihypertensive medicine, *p*-value results use cox proportional univariate, IDR = Indonesian Rupiah, BMI = body mass index.

**Table 3:** Prevalence of hypertension based on occupational factors in Karawang and Bogor in West Java, Indonesia 2018.

Variables	Total (n = 354)	% Hypertension (n = 165)	95% CI	*p*-Value
Duration of farming (years)				0.59
≥20	204	45.6	38.7–52.5	
15–19	43	41.9	26.5–57.2	
<15	107	50.5	40.8–60.1	
Farming methods				0.048
Modern	43	60.5	45.2 -75.7	
Mix (traditional and modern)	183	49.2	41.9–56.5	
Traditional	128	38.3	29.7–46.8	
Pesticides used				0.18
Yes	332	47.9	42.5–53.3	
No	22	27.3	7.1 -47.5	
Duration of pesticides’s used (year)				0.44
≥20	137	46.7	38.3–55.2	
10–19	96	53.1	42.9–63.3	
<10	99	44.4	34.5–54.4	
No	22	27.3	7.1–47.5	
Pesticide sprayer				0.065
Yes	322	48.8	43.3–54.2	
No	32	25.0	9.1–40.9	
Heat				0.018
High WBGT	186	54.8	55.1–69.9	
Low WBGT	168	37.5	30.1–44.9	

Definition of hypertension: averaged blood pressure was ≥140 mmHg systolic and/or diastolic ≥ 90 mmHg and/or they self-reported that they were currently taking antihypertensive medicine, *p*-Value results use cox proportional univariate, WBGT = The Wet Bulb Globe Temperature.

**Table 4:** Risk of hypertension, characteristics, lifestyle, and occupational factors among farmer in Karawang and Bogor in West Java, Indonesia 2018.

Variables	PR ^1^ (95% CI)	*p*-Value	Variables	aPR ^2^ (95% CI)	*p*-Value
Age (year)	0.99 (0.98–1.01)	0.69	Heat		0.037
Marital Status		0.53	Karawang/High WBGT	1.41 (1.02–1.95)	
Did not have spouse	0.81 (0.43–1.54)		Bogor/Low WBGT	1	
Have spouse	1		Pesticide sprayer		0.078
Educational level		0.76	Yes	1.90 (0.93–3.87)	
Low	1.09 (0.61–1.97)		No	1	
High	1		Smoking status		0.94
Income	1.01 (0.88–1.17)	0.81	Ever	1.02 (0.57–1.84)	
BMI (kg/m^2^)		0.406	Never	1	
25.0–≥30	1.18 (0.79– 1.76)		Alcohol consumption		0.41
<18.5–24.9	1		Ever	1.22 (0.76–1.97)	
Non-fasting blood sugar		0.92	Never	1	
High (≥200 mg/dL)	0.95 (0.35–2.57)		Age (year)	0.99 (0.98–1.01)	0.99
Normal	1				
Smoking status		0.97			
Ever	1.01 (0.56–1.82)				
Never	1				
Alcohol consumption		0.18			
Ever	1.37 (0.87–2.17)				
Never	1				
Red meat and high fat meat consumption		0.27			
Frequent	0.84 (0.62–1.14)				
Seldom	1				
Salted Fish or squid consumption		0.18			
Frequent	0.81 (0.59–1.09)				
Seldom	1				
Fruits and vegetables consumption		0.71			
Seldom	1.06 (0.78–1.43)				
Frequent	1				
Lenght of farming (years)	1.00 (0.99–1.01)	0.99			
Farming methods		0.048			
Modern	1.58 (0.98–2.54)				
Mix (traditional and modern)	1.28 (0.91–1.82)				
Traditional	1				
Lenght of pesticides’s used (year)	1.00 (0.99–1.01)	0.39			
Pesticide sprayer		0.065			
Yes	1.95 (0.96–3.97)				
No	1				
Heat Stress/Location		0.018			
Higher WBGT/Lower altitude/Karawang	1.46 (1.07–2.00)				
Lower WBGT/Higher altitude/Bogor	1				

^1^ PR: crude PR by cox regression univariate, ^2^ aPR adjusted PR (Final model), PR: prevalence ratio, CI: confidence interval.

## Data Availability

Not applicable.
